# Regulation of the innate immune cells during pregnancy: An immune checkpoint perspective

**DOI:** 10.1111/jcmm.17022

**Published:** 2021-10-28

**Authors:** Wen‐Xuan Li, Xiang‐Hong Xu, Li‐Ping Jin

**Affiliations:** ^1^ Shanghai Key Laboratory of Maternal‐Fetal Medicine Clinical and Translational Research Center Shanghai First Maternity and Infant Hospital School of Medicine Tongji University Shanghai China; ^2^ Shanghai Key Laboratory of Maternal‐Fetal Medicine Clinical and Translational Research Center Department of Biobank Shanghai First Maternity and Infant Hospital School of Medicine Tongji University Shanghai China

**Keywords:** immune checkpoint molecules, innate immune cells, pregnancy

## Abstract

The foetus can be regarded as a half‐allograft implanted into the maternal body. In a successful pregnancy, the mother does not reject the foetus because of the immune tolerance mechanism at the maternal‐foetal interface. The innate immune cells are a large part of the decidual leukocytes contributing significantly to a successful pregnancy. Although the contributions have been recognized, their role in human pregnancy has not been completely elucidated. Additionally, the accumulated evidence demonstrates that the immune checkpoint molecules expressed on the immune cells are co‐inhibitory receptors regulating their activation and biological function. Therefore, it is critical to understand the immune microenvironment and explore the function of the innate immune cells during pregnancy. This review summarizes the classic immune checkpoints such as PD‐1, CTLA‐4 and some novel molecules recently identified, including TIM‐3, CD200, TIGIT and the Siglecs family on the decidual and peripheral innate immune cells during pregnancy. Furthermore, it emphasizes the role of the immune checkpoint molecules in pregnancy‐associated complications and reproductive immunotherapy.

## 1 INTRODUCTION

The crosswalk between the mother and foetus during pregnancy using immunological markers remains enigmatic. The foetus has been first described as an allograft developing in an immunologically competent maternal host in 1953 by Sir Peter Brian Medawar.^1^ Various immunomodulatory cells are recruited into the placenta or proliferate locally, and residing in the decidua, as well as the cell surface receptors and secreted molecules, and are involved in the induction and maintenance of the tolerance throughout pregnancy.

The innate immune cells, such as the natural killer cells (NK cells), dendritic cells (DCs) and macrophages, are abundant at the early maternal‐foetal interface, where they differentiate into tolerogenic subsets with different functions.^2^, ^3^ The trophoblast cells selectively express a set of unique MHC molecules (HLA‐C, HLA‐E, HLA‐G), which allow simultaneous “loss‐of‐self” recognition and refusal to escape from the NK cells and macrophages through ligand‐binding, like the KIR family, NKG2 family, and LILR family.^4^, ^5^, ^6^

Immune checkpoint molecules are referred to as a series of co‐inhibitory receptors that are expressed on the immune cells regulating the degree of immune activation playing a positive role in inducing transplantation tolerance, tumour immune escape and autoimmune prevention.^7^, ^8^, ^9^ Some immune checkpoint molecules are observed on the innate immune cells during pregnancy playing a fundamental role in a successful pregnancy. However, the underlying mechanisms remain elusive. In this review, we investigated and summarized the current knowledge on the role of maternal‐foetal immunotolerance based on innate immune checkpoint molecules.

## 2 INNATE IMMUNE CELLS AT THE MATERNAL‐FOETAL INTERFACE

Immune cells are important components of the decidual microenvironment. A large part of the decidua comprises the maternal leukocytes (approximately 40%).^2^, ^3^ In the first trimester of pregnancy, the innate lymphoid cells (ILCs) with decidual NK cells (dNKs) comprise up to 70% of the decidual leukocytes, followed by the decidual macrophages (dMs) (20 to 25%) and T cells (3%–10%),^10^ indicating that the innate immune cells play an indispensable role in the maternal‐foetal interface.

### 2.1 Innate lymphoid cells

Five main ILC subsets have been identified in the decidua, including the dNK1–3, group 3 innate lymphoid cells (ILC3s) and proliferating NK cells.^11^ Unlike the peripheral blood NK cells and other tissue‐resident NK cells, the dNKs are have been typically defined as the CD56 superbright cells.^12^, ^13^ dNK1 was the most abundant subset identified in the cryopreserved ILCs (30%), followed by dNK2 (15%) and dNK3 (15%). dNK1 has larger granules, including perforin and granzyme, with higher expression of KIR, while dNK2 and dNK3 produce more cytokines.^11^ However, the origin of dNKs remains unclear. There are different pieces of evidence suggesting that dNKs are not only recruited by the CD56^bright^CD16^−^NK cells or by differentiation of CD56^dim^CD16^+^ NK cells in the peripheral blood but also from the immature NK precursors or mature hematopoietic CD34^+^ precursors in the uterus.^14^, ^15^, ^16^ DNKs can control the extravillous trophoblast invasion independent of a non‐cytotoxic mechanism and promote vascular remodelling. NK cells interact with and modulate other maternal immune cells. DCs can be primed by the activated NK cells, which inducing a protective CD8^+^ T‐cell response.^3^ The ILC3s are the RAR‐related orphan receptor gamma t^+^ (RORγt^+^) and secrete IL‐22, IL‐17A, IL‐8 and TNF‐α.^17^ In humans, two subsets of ILC3s have been classified as natural cytotoxic receptor‐negative (NCR^−^) and NCR ILC3s (also referred to as lymphoid tissue inducer (LTi)‐like cells). The NCR^+^ILC3s population is dominant in the human uterus and is involved in the recruitment and the survival of decidual neutrophils.^18^, ^19^

### 2.2 Macrophages

Generally, there are two macrophage populations: interferon‐γ (IFN‐γ) and lipopolysaccharide (LPS)‐induced proinflammatory M1 macrophages, and IL‐4‐induced anti‐inflammatory M2 macrophages in vitro. The circulating monocytes are precursors of the tissue macrophages. The dMs were identified as M2 macrophages.^20^ However, new data showed that the three macrophage subsets were identified in the uterus during early human pregnancy, CCR2^−^CD11c^LO^ (80%), CCR2^−^CD11c^HI^ (5%) and CCR2^+^CD11c^HI^ (10–15%). CCR2^+^CD11c^HI^macrophages are pro‐inflammatory, while CCR2^−^CD11c^HI^macrophages are anti‐inflammatory. The CCR2^−^CD11c^LO^macrophages showed the least inflammatory properties and appeared to be resting.^21^

### 2.3 Gamma delta T cells

A resident T‐cell population in the decidua is significantly enriched in the gamma delta T cells (γδTs) compared to the blood.^22^ The γδTs have been implicated in the responses to infectious diseases, in the regulation of immune responses, and in tissue homeostasis and repair.^23^ The percentage of γδT in the decidual CD3^+^ T cells is controversial.^24^ The decidual γδTs are large granular lymphocytes enriched in the cytoplasmic granules that do not express the CD4 or CD8 markers. The TCR of the decidual γδTs is composed of a V γ 9 / V δ 1 chain, which is different from the peripheral blood lymphocytes expressing the V γ 9 / V δ 2 chain.^25^ Early pregnancy can also induce the activated and terminally differentiated proinflammatory γδ T‐cell effectors to recruit at the maternal‐foetal interface with a variety of TCR components.^26^ These data confirm that the decidual γδT can suppress the population of the other cells and promote the proliferation and invasion of human trophoblast cells via IL‐10 and TGFβ.^24^, ^27^

### 2.4 Mucosal associated invariant T cells

The mucosal‐associated invariant T (MAIT) cells are a type of immune cell subset that is relatively rich in intervillous cells in the decidua and term pregnancy compared to the peripheral blood. The MAIT cells are anti‐microbial and tissue repair T cells, and cannot respond to the same HLA molecules, representing the ideal characteristics of the effector cells in the foetal mother interface.^28^ Decidual MAIT cells produced higher levels of granzyme B and similar levels of the IFN‐γ‐responsive *Escherichia coli* compared to the peripheral MAIT cells.^29^ The intervillous MAIT cells can interact with the foetal macrophages located in the injured regions of the villous tissue and promoting tissue repair.^30^

## 3 IMMUNE CHECKPOINT MOLECULES

The immune checkpoints are inhibitory pathways in the immune system and are regulated by the ligand/receptor interactions. They play an important role in maintaining the autoimmune tolerance and regulating the duration and amplitude of the physiological immune response to avoid damage and destruction of the normal tissues caused by the immune system. There are few reviews that focus on the area of immune checkpoints.^31^, ^32^ Although the immune checkpoint molecules were originally been identified in the T cells, the innate immune cells are becoming a newly emerging target. The most classic immune checkpoints are PD‐1 and CTLA‐4 represent the most common immune checkpoints. Some new molecules have been identified recently, including TIM‐3, LAG‐3 CD200, TIGIT and the Siglecs family.

PD‐1 is a 288 amino acid type I transmembrane protein composed of one immunoglobulin (Ig) superfamily domain, including a transmembrane domain, and an intracellular domain containing an immunoreceptor tyrosine‐based inhibitory motif (ITIM) and an immunoreceptor tyrosine‐based switch motif (ITSM).^33^ PD‐1 is found in a variety of immune cells, including the T cells, B cells and myeloid cells, NK cells, NKT cells and other congenital lymphoid cells.^34^, ^35^, ^36^ PD‐1 also resists TCR and CD28 positive signals by binding its ligand to the programmed cell death 1 ligand 1 (PD‐L1) and/or PD‐L2 leading to the T‐cell inhibition, which results in immune escape.^36^

CTLA‐4 is a co‐inhibitory receptor in many immune cells and interacts with the ligands B7‐1 (CD80) and B7‐2 (CD86). CTLA‐4 exerts its immunosuppressive effects by competing with its counterpart CD28.^37^ CTLA‐4 is recognized as a negative regulator of T‐cell activation and a regulator of the peripheral T‐cell tolerance and auto responsiveness. Regulatory T (Treg) cells inhibit other T cells by affecting APC (such as NK cells, DCs, and macrophages) function via CTLA‐4 and CD80/86 ligand‐binding rather than a direct connection.^38^, ^39^, ^40^, ^41^, ^42^, ^43^

Tim‐3 is a member of the Tim family and was first identified as a receptor expressed on the CD4^+^ and CD8^+^ producing IFN–γ.^44^ Tim‐3 consists of five atypical cysteine amino‐terminal immunoglobulin variable domains, a mucin stem, a transmembrane domain, and a cytoplasmic tail.^45^ The Tim‐3 cytoplasmic tail has no classical inhibitory signal transduction motifs, such as ITIM or ITSM.^46^ The expression of Tim‐3 is not limited to the T cells, but different types of immune cells, including the B cells, Tregs, NK cells, DCs, monocytes and macrophages are also expressed.^47^ Four ligands have been reported to bind to Tim‐3, including galectin‐9 (Gal‐9), phosphatidylserine, CEACAM1 and high mobility group protein B1 (HMGB1).^47^ The Tim‐3—galectin‐9 interaction plays a role in inhibiting immune response.

The lymphocyte activation Gene‐3 (LAG‐3) is a cell surface molecular protein encoded by the *LAG*‐*3* gene, which is mainly expressed in activated T cells, NK cells and plasma cell‐like dendritic cells. The main ligand of LAG‐3, MHCII, which negatively regulates the proliferation and activation of the T cells, is similar to that of CTLA‐4 and PD‐1. Fibrinogen‐like protein 1 (FGL1), a liver‐secreted protein, is a major LAG‐3 functional ligand‐independent from MHC‐II.^48^ It plays a role in inhibiting the function of the Treg cells and maintains the tolerance of the CD8^+^ T cells. LAG‐3 in the Treg cells is essential for the T‐cell exhaustion.^49^ Siglecs are a class of classical immunoglobulin‐like lectins and are comprising of 2–17 extracellular Ig domains, including an amino‐terminal V‐set domain that contains the sialic acid‐binding site. Most of the cytosolic domains of Siglecs have ITIM based on immune receptor tyrosine and generate negative signals by recruiting tyrosine phosphatases. Siglecs participate in discriminating between the self and non‐self by recognition of the sialic acid‐containing glycans in all the mammalian cells. Since Siglecs are expressed in most cells of the innate and adaptive immune system, they can control immune cell function by regulating the activated and inhibitory receptors.^50^, ^51^, ^52^

CD200 (OX‐2) is a member of the immunoglobulin (Ig) superfamily containing two extracellular Ig domains and an intracellular domain motif.^53^ CD200 is a protein present at the first line of the immune defence and was expressed on a large number of cell surfaces, including the B cells and activated T cells.^54^, ^55^ CD200R, the receptor of CD200, is also a member of the Ig superfamily. It is mainly expressed in the macrophages, microglia and DCs, as well as in the granulocytes and some subsets of T cells and B cells.^56^, ^57^ Activation of CD200R can inhibit the activity of the myeloid cell in vitro. Unlike most Ig superfamily members, CD200R lacks the ITIM domain, but it has a 67aa intracellular segment containing three tyrosine residues.^58^ After activation by CD200R, the third tyrosine will be phosphorylated, leading to the aggregation and phosphorylation of Dok‐1 and Dok‐2 with subsequent combination of RasGAP and Ship. In macrophages and rod cells, this reaction can inhibit the phosphorylation of ERK, JNK and p38. The mouse experiments have shown that the activation of CD200R in macrophages could inhibit the autoimmune response. These results suggest that the CD200‐CD200R signal is involved in the inhibition of the myeloid cell function.^58^, ^59^, ^60^, ^61^, ^62^

TIGIT was first identified as an immune checkpoint rheostat that suppresses the activation of the T cells using a bioinformatics algorithm.^63^ TIGIT was named differently, including WUCAM, and later changed to Vstm3 and then to TIGIT.^64^, ^65^, ^66^ TIGIT consists of an extracellular IgV region, a transmembrane domain, and a cytoplasmic tail that harbours a canonical ITIM and an immunoglobulin tail tyrosine (ITT)‐like phosphorylation motif.^63^ The expression of TIGIT was identified in the NK cells, effector cells and memory T cells and Treg cells.^63^, ^66^, ^67^ TIGIT binds two ligands CD155 and CD112, promoting an inhibitory signal and compete with the CD266 (DNAM‐1) or CD96, delivering a positive costimulatory signal. The ligands are expressed on APCs, T cells, and a variety of non‐hematopoietic cell types including the tumour cells.^63^, ^64^, ^67^, ^68^

### 3.1 The functions of the immune checkpoint molecules in normal pregnancy

The functions of the immune checkpoint molecules in innate immune cells during pregnancy are summarized in Table 1. During pregnancy, PD‐1 expression has been identified in a broad spectrum of decidual innate cell subsets including macrophages, NK cells, NKT‐like cells, ILC3, γδT cells and MAIT cells. DM polarization and function depend on the PD‐1 signalling during early pregnancy.^69^ The polarization of GM‐CSF‐differentiated macrophages towards the M2 phenotype can be promoted by PD‐1 agonist (PD‐L1 Fc), an activator of the PD‐1, which has higher phagocytic activity and can be reversed by the PD‐1 blockade via reprogramming metabolism, especially the strength of glycolysis. Indeed, glycolysis can be regulated by the PI3K/AKT/m‐TOR and MEK/ERK signalling. PD‐1 blockade is related to the favoured M1 macrophages as well as increased foetal resorption and a shift in the ratio of M1/M2 macrophages in an animal model by inhibition of the PD‐1 pathway, indicating a potential reason for pregnancy loss. When co‐cultured with the trophoblast cells, macrophages increase IFN‐β production through TLR4/LPS activation, which enhances the constitutive production of soluble PD‐L1 from the trophoblast cells. PD‐1 expressed on the macrophages interacts with the soluble PD‐L1 from trophoblast cells, which could induce the development of the M2 phenotype and consequently decrease inflammation.^70^ The soluble PD‐L1 has been shown to increase during pregnancy and may play a role in immune tolerance, which is critical for the foetus, which has already been confirmed.^71^, ^72^ Compared to the peripheral cells, the dNKs, dNKTs and decidual γ/δ T cells showed upregulated PD‐1 expression and reduced cytotoxic potential,^73^ especially the CD56^+^dNKs.^74^ The first evidence that human decidual ILC3s express a functional PD‐1 has been confirmed. Both CD56^+^ILC3s and LTi‐like cell subsets are suppressed function by PD‐1 during the first trimester of pregnancy, which decreases cytokine production including IL‐22, IL‐8, and TNF‐α. The embryonic trophoblast cells affect the activation of ILC3 by expressing high levels of PD‐L1, suggesting that PD‐1/PD‐L1 interaction may regulate ILC3 function at the foetal‐maternal interface. Notably, ILC3s decrease the PD‐1 expression significantly with the development of pregnancy. The results showed that the expression of PD‐1 on ILC3s (especially LTi‐like cells) is indirectly related to the pregnancy stage.^75^ The MAIT cells could play an important role in protecting the foetus from bacterial infections and maintaining homeostasis at the foetal‐maternal interface.^28^ The decidual MAIT cells expressed high levels of CD69, consistent with the tissue‐residency phenotype. MAIT cells expressed higher levels of PD‐1, CD38, and CD25 in the decidua parietalis than cells in the decidua basalis, indicating MAIT cells were more activated.^74^ However, intervillous blood MAIT cells are not activated but display a lower PD‐1 expression compared to the peripheral blood, which means they are highly armed to quickly respond if bacteria are encountered at the foetal‐maternal interface.^29^

**TABLE 1:** Summary of the function of the immune checkpoint molecules in the immune cells during pregnancy

Immune checkpoint molecules	Expression of receptor	Ligands	Expression of ligands	Biological function	Reference
PD‐1	Macrophages NK cells NKT‐like cells ILC3s γδT cells MAIT cells Activated T cells	PD‐L1	DCs DSCs Trophoblasts Macrophages NK Cells NKT‐like cells CD4^+^ T cells, Treg cells	Macrophage polarization: shift M1 to M2 reduce cytotoxic potential decrease cytokine production anti‐bacteria promote a shift to Th2 bias, Treg cell proliferation and T‐cell exhaustion	29, 69, 73, 74, 75, 76, 77, 78, 80, 82, 83, 84, 85, 86, 87, 88, 89, 90, 91, 92, 93
		PD‐L2	PMN‐MDSCs Neutrophils Syncytiotrophoblasts		
Tim‐3	Macrophages	Gal−9	Trophoblasts Treg cells	Macrophage polarization:shift M1 to M2	73, 75, 97, 98, 99, 100, 101, 102, 103, 104, 105
	Monocytes			Inhibition of cell function	
	NK cells			Increase the production of anti‐inflammatory cytokines	
	ILC3s Activated T cells			Reduce IL‐22 production regulate the balance of Th1 /Th2 and the effect of CD8 + T cells	
CTLA‐4	Treg cells Activated T cells	CD80 CD86	dNKs dmyelomonocytic cells	Improve IFN–γ and IDO in dNKs and dmyelomonocytic cells shifting of cytokines from Th1 predominance to Th2 bias T‐cell exhaustion	41, 89, 106, 107, 108, 109
Siglec‐7	Monocytes	GdA	Decidual cells	Induce the polarization of monocytes into dMs	116
Siglec‐10	Endometrial glands	CD24	Cytotrophoblasts	A possible role in mediating immune tolerance	117
	Decidual cells				
CD200R	Macrophages	CD200	Trophoblasts MSCs	Macrophage polarization: shift M1 to M2	62, 118, 119
TIGIT	γδT cells MAIT cells Activated T cells	CD155 CD112	APCs?	Regulate the cytotoxic activity increased tolerogenic properties inhibit cell proliferation and activation	126, 127
Lag‐3	Activated T‐cell	FGL1	APCs?	T‐cell exhaustion	87, 89, 90

PD‐L1 is expressed on the DCs, decidual stromal cells (DSCs), trophoblasts and even on the macrophages,^76^ CD4^+^ T, Treg, NKT‐like and CD56^+^ NK.^77^ Oestrogen (E2) increases PD‐L1 expression on APCs, including macrophages and DCs, and reduces the ability of the bone marrow‐derived dendritic cells (BM‐DC) to activate the T cells via *Esr1* signaling.^76^ Immunohistochemistry using an anti‐PD‐L1‐specific antibody demonstrated that in early and term normal placentas, PD‐L1 expression in syncytiotrophoblasts is higher than that in the intermediate trophoblastic cells.^78^ PD‐1 binding to its corresponding receptor PD‐L1, a costimulatory ligand expressed on dMs during the early pregnancy, can negatively regulate T‐cell activity. DMs can suppress the T‐cell IFN‐γ production via PD‐L1: PD‐1 interactions, whereas PD‐L1 was not expressed on the peripheral monocytes of the pregnant women.^79^ The levels of soluble PD‐L1 are elevated in the serum of pregnant women, which can suppress maternal immunity.^72^ Compared to peripheral blood, elevated PD‐L1 expression by CD4^+^ T, Treg, NKT‐like and CD56^+^ NK cells were also found with the increased PD‐1 expression by decidual CD8^+^ T, CD4^+^ T and NKT‐like cells.^77^ PD‐L2 is expressed on the decidual polymorphonuclear myeloid‐derived suppressor cells (PMN‐MDSCs) and neutrophils^76^, ^80^ and is also expressed in syncytiotrophoblasts.^81^ T‐cell proliferation is suppressed by both decidual PMN‐MDSCs and decidual explant supernatant (DES)‐conditioned neutrophils via PD‐1 signalling. A shift from circulating neutrophils to PMN‐MDSC‐like phenotypes requires stimulation with decidua‐derived granulocyte‐macrophage colony‐stimulating factor (GM‐CSF) by pSTAT5/PD‐L2 signaling.^80^ Overall, the PD‐1‐PD‐L1 axis not only regulates T cells including promoting a shift to Type 2 helper T‐cell (Th2) bias^82^ and Treg cell proliferation,^83^, ^84^ as well as reducing CD8^+^ T accompanied by NKG2D activating receptor,^77^, ^85^, ^86^, ^87^, ^88^, ^89^, ^90^ Th17^83^, ^91^ and TFH^92^, ^93^ responses but also plays an important role in innate immune cells for pregnancy maintenance.^94^

Similarly, in both murine and human pregnancies, Tim‐3 is not only expressed on many T‐cell subsets^82^, ^85^, ^95^, ^96^ but also on monocytes and innate lymphocytes in the peripheral blood or maternal‐foetal interface. Tim‐3 is strikingly upregulated in the peripheral monocytes of pregnant women with IL‐4/STAT6 signaling.^97^ TIM‐3 is expressed not only on the monocytes and granulocytes infiltrating the uterus but also on uterine macrophages and DCs, including their different subsets such as monocytic myeloid‐derived suppressor cells (M‐MDSCs) and granulocytic myeloid‐derived suppressor cells (G‐MDSCs). The blocking of Tim‐3 leads to a decrease in the phagocytic properties of uterine macrophages, leading to the inability to clear the apoptosis and dead cells of the uterus and increases local inflammation and foetal rejection.^98^ Tim‐3 are biased towards M2 activation rather than M1 polarization during early pregnancy.^98^, ^99^ In the murine pregnancy, Tim‐3^+^pNKs exhibit immunosuppressive functions, including increasing the production of anti‐inflammatory cytokines, decreasing the production of proinflammatory cytokines and impairing cytotoxic activity.^100^ The Tim‐3^+^ peripheral NK cells (pNKs) reduce foetal loss in abortion‐prone and NK cell‐deficient mice.^101^ Meanwhile, Treg cells showed higher levels of galectin‐9(Gal‐9) expression.^100^ Tim‐3^+^pNKs are stimulated by Gal‐9, leading to signalling by JNK and AKT. Moreover, high‐level production of anti‐inflammatory cytokines and the induction of Treg cells differentiation occur in pregnant pNKs.^102^ TIM‐3 expression was lower in the BALB‐c mice dNKs and dNKTs with higher relative receptor expression. These dNKs and dNKTs, which contributes to the mild inflammation needed for a successful pregnancy.^73^, ^102^, ^103^ The interaction between Gal‐9 secreted by the human trophoblast cells and Tim‐3 promotes pNKs to a dNK‐like phenotype. Tim‐3/Galecin‐9 partially inhibits the dNKs cytotoxicity towards trophoblasts by impairing the degranulation process.^103^ It has also been reported that Tim‐3 is expressed in the ILC3s and IL‐22 production by CD56^+^ILC3s can be inhibited notably via monoclonal antibody (mAb)‐mediated cross‐linking of Tim‐3.^75^ Tim‐3 is highly expressed on murine decidual γδT cells than on the peripheral blood, thereby regulating the cytotoxic potential and providing efficient protection against pathogens.^104^ Tim‐3 and PD‐1 co‐expression are more likely to regulate the balance of Th1 /Th2 and the effect of CD8^+^ T cells^82^, ^85^ as well as a part of CD56^+^ILC3s and LTi‐like cells.^75^ The release of soluble Tim‐3 from peripheral blood mononuclear cell (PBMC) cells into the circulation and its binding to the Gal‐9 may modulate Tim‐3‐mediated activity and help optimize immune regulation during pregnancy.^105^

In murine pregnancy, the abortion‐prone CBA/J mice treated with anti‐CD80 and anti‐CD86 mAbs exhibited a higher CD4^+^CD25^+^ Treg cell population and upregulated expression of CTLA‐4 and induced shifting of cytokines from Th1 predominance to Th2 bias.^106^, ^107^ Increased the CD4^+^CD25^+^ Treg cell population and upregulated *IDO* mRNA expression by using adenovirus‐mediated CTLA‐4 Ig gene transfer, which shifts the pregnancy outcome in a rat model of spontaneous abortion.^108^ In human pregnancy, the production of IFN‐γ and the expression of IDO are improved by the interaction between dNKs and decidual CD14^+^ myelomonocytic (dmyelomonocytic) cells, which increases the induction of the Treg cells. The CD14^+^ dmyelomonocytic cells may affect the Treg cells by TGF‐β production or CTLA‐4 mediated interaction.^41^ The CD4^+^CD25^+^ CTLA‐4i^+^ decidual Treg cells inhibit the proliferation of other T cells by anti‐CD3 stimulation^109^

The exhausted CD4^+^ T cells increase in the decidua parietalis with the progression of the gestational age, whereas these cells decrease in the presence of placental inflammation in the decidua basalis of women in preterm labour. Moreover, the decidual exhausted T cells produce the IFN*γ* and TNF*α* upon in‐vitro stimulation.^110^ Other studies have found that the CD69^+^ CD103^+^ resident memory T cells were abundant in the CD8^+^ decidual T(dT) cells but not in the CD4^+^ dT cells with effector‐memory phenotypes. Compared to the peripheral blood, the tissue‐resident molecules CD103 and CXCR3 are increased significantly with almost no perforin secretion in the CD8^+^dT cells, indicating that the decidual microenvironment is essential for the residency of the CD8^+^T cells.^86^ Furthermore, these cells showed high expression of the chemokine receptors, T‐cell exhaustion‐related molecules, and co‐inhibitory molecules PD‐1, CTLA‐4 and LAG3.^86^, ^87^, ^89^, ^111^ Moreover, they produced more anti‐inflammatory cytokines and effector cytokines after stimulation.^111^ The tissue‐resident molecules CD103 and CXCR3 were increased significantly with almost no perforin secretion in the CD8^+^dT cells compared to the CD8^+^pT cells. Importantly, the decidual T‐cell function can be restored upon in‐vitro stimulation, indicating that the CD8^+^dT cells are not permanently suppressed and retain the capacity to respond to proinflammatory event.^89^, ^110^ The T‐box transcription factor protein eomesodermin (Eomes) is beneficial for maintaining the homeostasis and function of the effector and memory CD8^+^T cells that show increased expression during pregnancy.^112^ The pregnancy‐primed maternal CD8^+^ T cells were expanded as a distinctive memory pool, and also showed an enriched expression of negative costimulatory molecules, including PD‐1 and LAG‐3 after parturition, while CTLA‐4 and TIM‐3 levels did not change significantly.^90^, ^113^ Consistent with primary pregnancy, the expression of PD‐1, LAG‐3 and CTLA‐4 was increased by maternal CD8^+^T cells with foetal re‐stimulation during secondary pregnancy, whereas the expression of TIM‐3 remained unchanged. Fatal wastage was induced by PD‐L1/LAG‐3 functional blockade with the activation of foetal‐specific CD8^+^T cells during secondary pregnancy. Collectively, these findings indicate that the CD8^+^T‐cell functional exhaustion maintains foetal tolerance during primary pregnancies compared to the subsequent pregnancies.^90^

Sialic acid (Sia) is dispensable for protecting the foetus from maternal complement attack.^114^ Moreover, Sia can combine with Siglecs as a ligand, which is critical or restraining the immune response.^50^ The ability of the trophoblast invasiveness is suppressed by the interaction between the Siglec‐6 and Glycodelin‐A protein (GdA), a glycoprotein that is abundantly expressed in the decidua and participates in defence and placental development, which downregulating the ERK/c‐Jun signalling pathway.^115^ Siglec‐7 mediates the binding of the GdA in a sialic acid‐dependent manner to induce the polarization of the monocytes into dMs to regulate immune tolerance and placental development.^116^ Co‐localization of Siglec‐10 with CD24 observed at the maternal‐foetal interface especially in the endometrial glands and decidual cells close to extracellular trophoblasts suggests a possible role in maintaining immune tolerance.^117^

In murine pregnancy, the expression of CD200 is expressed in the decidual cell subsets and trophoblasts of the mouse uterus.^118^ Embryo resorption is inhibited by CD200 expression in the trophoblasts.^118^ Macrophages and mast cells also express CD200R. Upregulation of CD200 alters DCs to promote Treg cell development and suppress the macrophages and mast cells.^119^ Mesenchymal stem cells (MSCs) also express CD200 and the interaction with CD200R on the decidual M1 and CD200 on MSCs promoted the shift of the proinflammatory macrophage phenotype to an anti‐inflammatory phenotype.^120^ The limited expression of CD200R subtypes at the maternal‐foetal interface suggests that the interaction of CD200 and CD200R may play an important role in determining the successful outcome of pregnancy.^121^ In human pregnancy, compared to the non‐pregnant women, the population of peripheral blood monocytes in early pregnant women is increased while the total number of leukocytes and lymphocytes show no significant changes. These monocytes are found to be upregulated the expression of CD200 and CD200R.^122^ CD200R can regulate the cytotoxic activity of the NK cells during human pregnancy.^123^ The expression of CD200 and CD200R on the CD1c^+^myeloid and BDCA‐2^+^ lymphoid DCs in the first trimester of normal pregnancy was significantly higher than that in the non‐pregnant women, suggesting that the myeloid and lymphoid DCs increased tolerogenic properties in early pregnancy.^124^ After 5 weeks of gestation, the CD200 tolerance signal molecule and its receptor CD200R1 were expressed in the human placental villous trophoblasts and implant decidua.^125^

In the peripheral blood, TIGIT is expressed on the γδT cells and MAIT cells.^126^, ^127^ The decidual γδT cells and Treg cells also can also express TIGIT.^126^, ^128^ After blocking the TIGIT antibody, the γδT cells can induce proliferation and secrete an immunomodulatory protein called progesterone‐induced blocking factor (PIBF), which can facilitate immune escape and is important in preventing embryonic rejection. These results indicate that TIGIT pathways are important for maintaining immune tolerance.^126^

### 3.2 The roles of the immune checkpoint molecules in pregnancy complications

#### 3.2.1 Recurrent Spontaneous Abortion (RSA)

Recurrent Spontaneous Abortion is a pregnancy complication evident in 1% of pregnant females characterized by heterogeneous nomenclatures such as recurrent pregnancy loss (RPL) and recurrent miscarriage (RM). According to guidelines of the European Society for Human Reproduction and Embryology (ESHRE) and the American Society for Reproductive Medicine (ASRM), RSA is defined as two or more consecutive spontaneous abortions.^129^, ^130^, ^131^ The pathogenic factors of RSA have been identified by either genetic, structural, infective, endocrine, immune or unexplained causes.^129^ Immune abnormalities are the root cause of pregnancy complications.^132^

**FIGURE 1:**
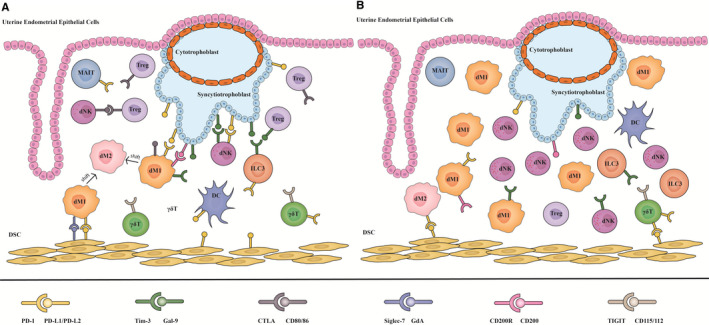
Roles of the immune checkpoint molecules in normal pregnancy and RSA. (A) In normal pregnancy, the expression of immune checkpoint molecules was identified in a broad spectrum of decidual innate immune cells, such as PD‐1, which is expressed on the dMs, dNKs, ILC3, γδT cells, MAIT cells; Tim‐3 is expressed on the dMs, dNKs, ILC3; Siglec‐7 and CD200R are expressed on the dMs; TIGIT is expressed on the γδT cells, MAIT cells and the related ligands are more expressed on the DSCs and trophoblasts. These co‐inhibitory receptors maintain the maternal‐foetal immunotolerance, which promotes the macrophage polarization to dM1and reduces the dNKs cytotoxic potential. (B) In RSA, while the population of the anti‐inflammatory cells (like Treg cells and γδT cells) decrease and γδT cells upregulate the expression of PD‐1 and TIGIT, the proinflammatory cells increase and downregulate the expression of the immune checkpoint molecules

The roles of the immune checkpoint molecules in RSA compared to that in the normal pregnancy (NP) are shown in Figure 1. The percentage of dM1 was significantly higher in the RSA. Similarly, the M2 population was lower in the RSA than in the NP. The data confirmed the expression of PD‐1 and PD‐L1 in both dM1 and dM2, which showed a significant difference between NPs and RSA. Overall, RSA had lower PD‐1 expression in dM1 than in NP and dM2. Although PD‐L1 is more in the dM1 than in dM2, RM decreases the percentage of PD‐L1^+^ dM2 compared to NP.^70^ In RSA patients, PD‐1 expression in the γδT cells in the decidua increases significantly, whereas, in the peripheral blood, it does not change. In addition, co‐culture with progesterone, TIGIT and PD‐1 blocking antibodies increased γδT‐cell proliferation and progesterone‐induced blocking factor (PIBF) production in the culture medium supernatant significantly. These results indicate that the TIGIT and PD‐1 pathways are important in maintaining immune tolerance.^126^ The pNKs reduce Tim‐3 expression and immunosuppressive function in patients with RSA. The Tim‐3‐Gal‐9 signalling‐mediated immunoregulation by pNKs plays a critical role in maternal‐foetal immune tolerance and suggests that Tim‐3 abundance on pNKs is a potential biomarker for RSA diagnosis.^101^ Clinical data show that abnormal Tim‐3 levels on pNKs might be associated with RSA^103^ and interference of cytokine profiles and increased cytotoxicity are observed in the Tim‐3^+^dNKs.^102^ In human miscarriages and murine abortion‐prone models, dNKs decrease the percentage of Tim‐3‐expression and the production of the Th2‐type cytokines while Th1‐type cytokines are increased.^102^ DSCs decrease the expression of Tim‐3 in the miscarried women who miscarried. Tim‐3 is a key regulator of DSCs and s shows a potential role as a target for the treatment of spontaneous abortion.^133^ Proinflammatory cytokine production and cell apoptosis in DSCs are induced by the activation of TLR signalling, which could be inhibited by Tim‐3 in the ERK1/2 pathway‐dependent manner and suppressing of NF‐κB activation.^133^ The *CTLA*‐*4* gene is first involved in maintaining immune tolerance at the maternal–foetal interface and plays a role in inducing RSA.^134^ The surface density of CTLA‐4 on decidual CD26^+^lymphocytes was lower in the model of stress‐induced abortion and returned to normal levels by using inhibiting of DPP IV.^135^ The proportion of decidual CD4^+^CD25^+^ T cells was significantly lower.^109^ The CD4^+^CD25^+^ Treg cells increase the induction of IFN‐y to upregulate the IDO expression in DCs and monocytes in normal pregnancy, but downregulate in RSA.^136^ CD200 and its receptor CD200R1 decrease at 7–9 weeks of gestation in RSA.^125^ CD200‐CD200R1 signalling may be required for successful human pregnancy. The population of γδT cells in the decidua shows a significant decrease. These decidua γδT cells also upregulate the expression of TIGIT while the peripheral γδT cells did not change significantly.^126^

The number and function of Tim‐3^+^ PD‐1^+^ CD8^+^ T cells in the decidua were significantly suppressed in RSA.^85^ Patients with RSA show a lower expression of PD‐1 and PD‐L1 on Th17 cells, as well as Th1 cells, which may lead to increased Th1 and Th17 immune responses, and an imbalance between the Th17, Th1 and Treg cells in the women with RSA.^137^ Compared to patients with negative antibodies, decidual CD4^+^ CXCR5^+^ PD‐1^+^ ICOS^+^ memory Tfh cells were significantly increased in RSA patients with positive antibodies.^138^ The Treg cells decrease in the decidua in RSA, with no difference between the percentage of PD‐1 positive cells in decidual lymphocytes and that in the normal pregnant women. In addition, there was no change in Treg cell percentage when PD‐1 blockade was used, while the percentage of CD4^+^ T cells increased. The decrease in PD‐L1 may be closely related to the occurrence of RSA, and the suppression of Treg cell function in RSA may be due to PD‐L1 downregulation in decidua or trophoblast cells rather than PD‐1 direct change.^139^ An increase in the Tim‐3 expression in PBMCs and placental tissue of RSA might affect maternal‐foetal immune tolerance.^140^ Lower Tim‐3 expression was observed in CD4^+^ T cells and Treg cells in women with RSA, and the abnormal expression of IL‐27 and Gal‐9 is associated with impaired immunological tolerance in the RSA patients.^95^ The Treg cells and levels of CTLA‐4 and TGF‐β1 are decreased and the levels of IL‐6 and TNF‐α and the Th17/Treg ratio, are increased at the maternal‐foetal interface, leading to disordered maternal‐foetal immune tolerance in RSA.^138^, ^141^, ^142^ In RSA patients, the expression levels of CD49b and LAG‐3 on CD14^+^ monocytes and the plasma levels of TGF‐β were lower than those in the normal females.^143^

The percentage of decidual resident memory T cells in RSA patients was significantly higher than that in patients with normal pregnancy,^111^ and a decrease in circulating Tregs and exhausted CD8^+^ T cells was observed in the peripheral blood of RIF patients compared to the controls, whereas the exhausted Treg and Th17 cells were more frequent.^144^, ^145^ and the Eomes^+^dCD8^+^ T cells also decreased in RSA.^112^

#### 3.2.2 Preeclampsia (PE)

Preeclampsia occurs with gestational hypertension and proteinuria significantly in the third trimester, leading to serious complications, including maternal and foetal death.^146^ The innate immune cells participate in vascular remodelling and placenta development, which are central to the pathogenesis of this syndrome.

Except in the case of RSA, not only PD‐1 and NKG2D are significantly upregulated in the peripheral NKT cells^147^ but also the soluble PD‐1 levels are significantly increased in women with early‐onset PE.^148^ Lower expression of PD‐L1 and PD‐L2 proteins on peripheral monocytes in PE might indicate a secondary regulatory mechanism in response to the ongoing systemic maternal inflammation.^149^ In the LPS‐induced PE mouse model, DMs decreased the expression of Tim‐3, and the maternal‐foetal interface reduced the expression of Gal‐9. The population of dM1 is greater than dM2 in the PE model, which could be reversed by the administration of Gal‐9 protein.^99^ Upregulation of Tim‐3 in DMs and Gal‐9 at the maternal‐foetal interface suggests that the Tim‐3/Gal‐9 pathway may play an important role in early pregnancy and embryonic development. The Siglec‐6 expression promotes proliferation in a leptin‐dependent manner, but protects the cells from apoptosis and promotes invasion in a leptin‐independent manner.^150^ Differential expression of Siglec‐6 in invasive cytotrophoblasts, which is unique in spontaneous PE.^151^ The DCs decreased the CD200R expression.^124^, ^152^ There is a higher risk of PE to inhibit PD‐L1 and CD200, which suppresses the IDO expression in the placenta.^153^ The peripheral MAIT cells expressing TIGIT and CD226 immune checkpoint molecules have a marginal role in the pathogenesis of early‐onset PE. MAIT cells markedly differed in proportion, TIGIT expression, granzyme B and perforin content compared to the MAIT‐like and non‐MAIT cells.^127^ Siglec‐9^+^ CD16^+^/CD56^dim^ and CD16^−^/CD56^br^ NK cells increased binding of MUC16 was observed in the preeclampsia cohort as compared to the healthy pregnant samples, which contribute to tolerance of the foetal allograft from the maternal responses and may also serve as a novel biomarker for PE.^154^

Patients with PE showed fewer Treg cells, as well as a significant increase in the percentage of exhausted PD‐1^+^ Tregs compared to the normal group, indicating that it may be as a therapeutic target for the disease.^155^, ^156^ Moreover, decreasing PD‐1 expression might contribute to a higher Th17 cell frequency by promoting proliferation in PE.^91^ Patients with PE showed lower Tim‐3 expression on peripheral CD8^+^T cells that affected uterine spiral artery remodelling slightly.^157^

## 4 IMMUNE CHECKPOINTS IN REPRODUCTIVE IMMUNOTHERAPY

Abortion is the most common complication associated with pregnancy and RSA affects 1% of couples.^129^ Although different regions have different definitions,^158^ there is a common view of the mechanism of immune‐mediated RSA.^132^ Immunotherapy has proven to be effective for RSA, including administration of progesterone, cyclosporin A (CsA) and intravenous immunoglobulin (IVIG).^158^, ^159^, ^160^, ^161^

Progesterone plays an important role in the establishment and maintenance of pregnancy in an endocrine and immunological manner, which mainly depends on affecting cytokine synthesis and the function of the NK cells.^159^, ^162^ It can also treat RSA by restoring several co‐inhibitory molecules, including Tim‐3, PD‐1 and CTLA‐4.^163^ CsA is a useful immunosuppressant and is used to prevent allograft rejection. In addition to regulating the trophoblast growth and invasion,^164^, ^165^ CsA also contributes to maintaining the immunity tolerance at the maternal‐foetal interface by upregulating the expression of CTLA‐4 and downregulating the levels of CD80, CD86 and CD28.^166^ IVIG affects the outcome of RSA through several potential mechanisms. IVIG can regulate cytokines to promote the anti‐inflammatory effect and decrease the ability of pNKs in cytotoxicity.^158^ CD200 tolerance‐promoting signalling has been found to play a role in the suppression of pNKs by IVIG.^123^ IVIG increases the Treg cells and reduces the exhausted Treg responses in RM patients with abnormal cellular immunity during pregnancy. The immunomodulatory effects of IVIG may be related to pregnancy outcomes.^167^

## 5 CONCLUSIONS

Innate immune cells are the largest population of decidual leukocytes and play a critical role in reproduction.^3^ On the one hand, they resist the threat of foreign microbes and viruses to a healthy pregnancy, while on the other hand, they play a role in the trophoblast invasion and spiral artery remodelling, controlling embryo implantation and normal development of the placental bed. Meanwhile, a part of the innate cells, such as APC, connect with T cells and suppress the immune response to maintain a normal immunological microenvironment at the maternal‐foetal interface.^168^, ^169^ However, it is still not clear whether the innate immune cells are activated or inhibited by crosstalk with other cells during pregnancy complications, such as preeclampsia and recurrent spontaneous abortions. Many studies have shown that innate immune cells are regulated by the expression of immune checkpoint molecules via ligand‐binding, which may lead to a different perspective while investigating related mechanisms.

In recent years, immune checkpoint molecules have been actively researched worldwide because of their important roles in regulating the immune responses in health and disease. An increasing number of molecules have been found in immune tolerance, and exploring additional immune checkpoint molecules is a hot research topic, including their antibodies and the immune related adverse events.^170^, ^171^ In addition to the classical checkpoints such as PD‐1 and CILT4, we have also discussed several novel immune checkpoint targets that have recently been identified, such as TIM‐3, TIGIT, CD200R and Siglec. Although the studies are not abundant, they illustrated the importance and diversity of regulation in innate immune cells, indicating broader.

Recently, a series of soluble immune checkpoints have been identified, such as the soluble CTLA‐4 and soluble PD‐1, which can be diffuse in the serum. The soluble immune checkpoint molecules are functional parts of the membrane produced in different ways and can be secreted by the immune cells. Immune regulation can be controlled and changed by the plasma levels of the soluble immune checkpoints, which can control the development, prognosis and treatment of diseases.^172^ There is evidence that soluble PD‐1 and soluble Tim‐3 increase during pregnancy^70^, ^71^, ^72^, ^105^, ^148^, ^173^ and affect the immune regulation, which may play significant roles in pathogenesis, immune responses, and prediction of pregnancy outcomes. The soluble immune checkpoint may provide new biomarkers and potential insights into the immunotherapeutic strategies for pregnancy complications, such as RSA and PE.

Immune checkpoint inhibitors have changed the landscape of cancer treatment in the advanced stage. The National Comprehensive Cancer Network guidelines advise that patients of reproductive age use effective birth control during and for at least 5 months after immunotherapy. Most clinical trials also required patients of reproductive age to use at least two anticonception methods while receiving anti‐PD‐1 or anti‐PD‐L1 agents up to 6 months after the last dose. However, data supporting this recommendation are lacking.^113^ In the animal models or in vitro experiments, foetal loss or other pregnancy complications can be exacerbated by using antibodies related to the immune checkpoint molecules.^174^, ^175^ Anti‐PD‐1 administration increases the risk of spontaneous abortion in animal studies and is categorized as category D by the Food and Drug Administration, whereas ipilimumab is pregnancy category C.^176^ There is a unique case of conception and pregnancy. Both the mother and the foetus have been successful in the treatment of metastatic melanoma with anti‐CTLA‐4 and anti PD‐1 therapy.^177^ In order to understand the full effects of these drugs, long‐term follow‐up is needed, including other fertility surrogate indicators, such as subsequent pregnancy, abortion, live birth and birth defects.^113^ Reproductive safety must be considered when using blocking antibodies during pregnancy.

As mentioned above, the crosstalk between the mother and foetus during pregnancy remains an enigma. With the recognition of innate immune cells recognized, we speculate additional investigations and studies on the immune checkpoint molecules at the maternal‐foetal interface might soon be conducted.

## CONFLICT OF INTEREST

The authors have declared no conflict of interest.

## AUTHOR CONTRIBUTIONS

**Wen‐Xuan Li:** Conceptualization (equal); Data curation (equal); Visualization (equal); Writing‐original draft (equal); Writing‐review & editing (equal). **Xiang‐Hong Xu:** Conceptualization (equal); Supervision (lead); Writing‐original draft (equal). **Li‐Ping Jin:** Conceptualization (equal); Funding acquisition (lead); Supervision (lead); Writing‐original draft (supporting); Writing‐review & editing (equal).

## Data Availability

Data sharing is not applicable to this article as no new data were created or analysed in this study.
